# The Ground Effect in Anguilliform Swimming

**DOI:** 10.3390/biomimetics5010009

**Published:** 2020-03-03

**Authors:** Uchenna E. Ogunka, Mohsen Daghooghi, Amir M. Akbarzadeh, Iman Borazjani

**Affiliations:** 1 J. Mike Walker ’66 Department of Mechanical Engineering, Texas A&M University, College Station, TX 77843, USA; uchenna@tamu.edu (U.E.O.); daghooghi@uhcl.edu (M.D.); amirmahd@tamu.edu (A.M.A.); 2 Mechanical Engineering Program, University of Houston–Clear Lake, Houston, TX 77058, USA

**Keywords:** fish locomotion, eel swimming, ground effect, self-propelled, simulation

## Abstract

Some anguilliform swimmers such as eels and lampreys swim near the ground, which has been hypothesized to have hydrodynamic benefits. To investigate whether swimming near ground has hydrodynamics benefits, two large-eddy simulations of a self-propelled anguilliform swimmer are carried out—one swimming far away from the ground (free swimming) and the other near the ground, that is, midline at 0.07 of fish length (*L*) from the ground creating a gap of 0.04L. Simulations are carried out under similar conditions with both fish starting from rest in a quiescent flow and reaching steady swimming (constant average speed). The numerical results show that both swimmers have similar speed, power consumption, efficiency, and wake structure during steady swimming. This indicates that swimming near the ground with a gap larger than 0.04L does not improve the swimming performance of anguilliform swimmers when there is no incoming flow, that is, the interaction of the wake with the ground does not improve swimming performance. When there is incoming flow, however, swimming near the ground may help because the flow has lower velocities near the ground.

## 1. Introduction

Some aquatic swimmers such as eels and stingrays typically swim near the ground [1,2]. Eels are one of the anguilliform swimmers, which tend to swim closer to the ground in rivers and shallow streams [3]. They propel themselves by creating backward traveling waves, whose oscillations are parallel to the ground. In contrast to the eels, stingrays, one of the rajiform swimmers, have disk-shaped bodies and they propel themselves by oscillating their bodies normal to the ground. The ground effect has been studied for rajiform swimming [2], but not for anguilliform swimming.While the interaction of the wake and ground can enhance the cruising speed and decrease the cost of transport in rajiform swimming [2,4,5,6,7,8], it is not clear if it has hydrodynamic benefits in anguilliform swimming in which the oscillations are parallel to the ground.

In addition to aquatic swimmers, ground effect for oscillations along ground normal has been studied in relation to the aerodynamics of insects [9], and the hydrodynamics of oscillating foils [10,11,12,13,14,15]. To understand the role of wake-ground interactions on the hydrodynamics, several studies have been performed on foils with oscillations normal to the ground. For example, Quinn et al. [10] discovered that the fins of a flexible oscillating foil created unsteady wakes that could improve thrust. Kurt et al. [15] observed that the thrust of the free swimming foil increased closer to the ground by about 4%–17%. Dai et al. [13] also observed that the cruising speed of a flexible foil and its propulsion efficiency increases due to the ground effect. In addition, the wake behind the foil transitioned from a reversed Karman vortex street into a deflected vortex dipole street [13] when it undulates close to the ground. Nevertheless, all of these studies are focused on undulations normal to the ground, not parallel to the ground like eels’ undulations. In fact, to best of our knowledge, this study is the first study that investigates the ground effect for eels’ swimming.

In this paper, to quantify the ground effect in anguilliform swimming, we compute and compare the hydrodynamics of two anguilliform swimmers, that is, near ground swimming and free swimming, using self-propelled large eddy simulations (LES). There are two main mechanisms for hydrodynamic benefits of swimming near ground—(1) the ground can interact with the wake of the swimmer [15], or (2) the swimmer can benefit from swimming against a flow with a lower momentum. In a river or channel, the flow near the ground has a lower velocity (momentum) compared to the flow far from the ground. To eliminate the second mechanism and only consider ground-wake interaction, simulations are performed in an initially quiescent flow (no incoming flow) –see the methods (Section 2) for details. The velocity, thrust, and required power of anguilliform swimming near ground are compared against those of a freely swimming one (Section 3). The observed trends are explained by visualizing the flow field using velocity vectors and vorticity (Section 3). Finally, the results are discussed and the conclusions are reported in Section 4.

## 2. Materials and Methods

In this section, the fish kinematics is described in Section 2.1, the numerical method is presented in Section 2.2, the computational details are explained in Section 2.3, and the formulations for calculating hydrodynamic forces and efficiency are provided in Section 2.4.

### 2.1. Fish Body Kinematics and Non-Dimensional Parameters

**Figure 1:**
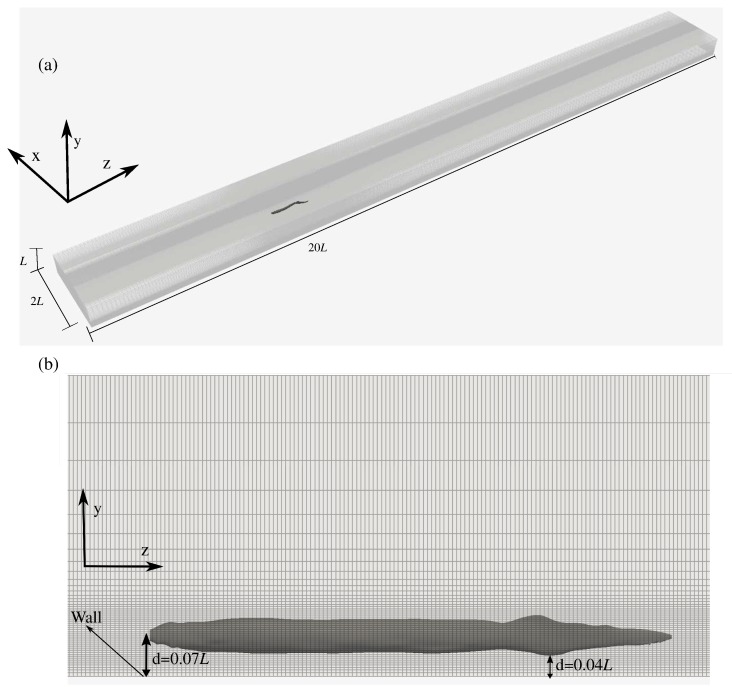
Eel geometry within computational grid; (**a**) 3D view, (**b**) two dimensional view with ground. Every other grid point is shown for better visualization.

The geometry of the swimmer is presented in Figure 1. The anguilliform swimmer swims along *z* direction by creating undulatory motions along *x* direction (Figure 1). The kinematics for anguilliform swimmers is usually in the form of a backward traveling wave, with the wave amplitude increasing almost linearly from the head to the tail of the fish [16]. The equation which describes the lateral undulations of the fish body is given below (all lengths are nondimensionalized with the fish length *L*):

(1) h(z,t)=a(z)sin(kz−ωt),

where *z* is the axial flow direction measured along the fish axis from the tip of the fish’s snout; h(z,t) is the lateral excursion of the body at time *t*; k=2π/λ is the wave number of the body undulations, where λ is the wavelength; ω=2πf is the angular frequency, where *f* is the tail beat frequency. Here, $a\left(z\right)=a_{max}e^{\alpha (z-1)}$ is the amplitude envelope of lateral motion [1], where α=2.18, $a_{max}$ is the maximum amplitude at the tail (z=L). Here, $a_{max}$ = 0.1*L* is within the range observed in previous experiments i.e., 0.05*L*–0.2*L* [1,17]. Two important similarity parameters in fish swimming are the Reynolds number and the Strouhal number. The Reynolds number Re=LU/ν is 40,000, based on the fish length (L=14 cm), the steady inline swimming speed (U=2L/s), and the water kinematic viscosity ($\nu =9.8\times 10^{-7}$ m${}^{2}$/s). The Strouhal number is $St=2a_{max}f/U$, based on the maximum lateral excursion of the tail along *x* direction (Figure 1) ($2a_{max}$). Here, the wavelength is 0.65*L*, and St is 0.5 as the anguilliform swimmers tend to swim at a high Reynolds number value of order $10^{4}$ [1,17,18,19,20] with a Strouhal number of 0.3–0.5 [18,21] and a body wavelength of 0.6*L*–0.65*L* [22].

### 2.2. The Numerical Method

The self-propelled anguilliform swimming is simulated by fluid-structure interaction (FSI) simulations [23,24]. In FSI simulations, the swimming velocity is calculated based on the hydrodynamic forces acting on the anguilliform swimmer using Newton’s second law of motion [23]. The simulation is continued until the anguilliform swimmer has a mean steady velocity. The hydrodynamic forces are computed by solving the flow around the fish which is governed by unsteady 3-D filtered incompressible Navier-Stokes in a non-inertial frame of reference [25,26]. The frame of reference is attached to the center of mass of the fish to reduce the required domain size and consequently the simulation costs. The unsteady 3-D incompressible Navier Stokes equation is discretized with central scheme and integrated in time using a second-order fractional methodology [24,27]. The momentum equations are solved with a Newton-Krylov solver and the pressure Poisson equation is solved with a generalized minimal residual method (GMRES) [28] enhanced with a multigrid as a pre-conditioner using the parallel libraries of portable, extensible toolkit for scientific computation (PETSc) [29].

The wake of the flow over bluff bodies becomes turbulent at Re>O(1000). At a high Re of 40,000, the wake of the swimmer becomes turbulent, whereas its boundary layer remains laminar. Therefore, the turbulent flow around the fish is modeled using a dynamic Smagorinsky LES modeling [30] which has been used and validated for simulating moving boundaries within turbulent flows [31,32]. The no-slip boundary condition on the moving boundaries of the swimmer’s body is handled using the sharp-interface immersed boundary method [24,27]. The numerical method has been implemented in our in-house code which is validated extensively for flows with moving boundaries and has also been applied successfully to simulate fish-like swimming [25,26,33,34,35].

### 2.3. Computational Details

The computational domain is a cuboid of 2L×L×20L along *x*, *y*, and *z* directions, respectively, for the free swimmer and 2L×0.576L×20L for the near ground swimmer. The free swimmer’s domain width and height are the same as the previous studies [23,36], but its length has increased about by three times. The domain of the free swimmer is discretized with about ≈14.6 million grid nodes, i.e., there are 201, 121, and 601 grid nodes along *x*, *y*, and *z* direction, respectively. A uniform mesh with constant spacing 0.004*L*, 0.002*L*, and 0.008*L* along the *x*, *y*, and *z* directions, respectively, is used to discretize an inner cuboid with dimensions 0.4*L*, 0.1*L*, and 1.4*L* enclosing the fish. The grid spacing along the oscillation direction *x* corresponds to wall normal spacing $x^{+}=u_{\tau }\delta x/\nu \approx 5.5$, where $u_{\tau }$ is the friction velocity and δx is minimum grid spacing along x direction. This wall normal spacing is enough to resolve the viscous sub-layer ($x^{+}<11.6$ [37]) of the swimmer’s boundary layer. This grid spacing of the inner cuboid of the free swimmer is the same as our previous study for fish locomotion [25], in which the turbulence modeling and Re were similar. To investigate the effect of ground, a second grid with the same grid spacing as the first one is created. The second domain is discretized with about 11.7 million grid points, that is, the number of grid points along *x* and *z* directions are the same as the first grid. However, the *y* direction of the domain is discretized with 97 points (104 fewer points than the free swimming case) because of the smaller domain size due to the presence of ground. The midline of the fish is placed at the vertical distance of 0.07*L* from the ground, which creates a gap of 0.04*L* as shown in Figure 1b. The grid spacing of the gap between the fish and ground remains constant 0.002L. The fish head is placed 5*L* from the boundary in the axial direction and centered in the transverse direction for both cases.

The tail beat period *T* is divided in 300 time steps, that is, δt=T/300. The results of previous simulations for fish locomotion [25,26] with similar Re, turbulence modeling, gird spacing and time step were shown to be grid and time insensitive. Therefore, the grid spacing and time step are sufficiently small, that is, results are grid and time step insensitive. Far-field (Neumann) boundary conditions with a correction to satisfy the mass conservation are applied on the domain boundaries of the free swimmer case, and for the near ground case, a no slip boundary condition is applied on the ground boundary, and the other boundaries are far-field boundary conditions, the same as the free swimmer case.

Here, flow is at rest at the initial time to see how the interaction of ground on the wake of the swimmer affects its hydrodynamics. The ground effect can influence the hydrodynamics of the near ground swimmer by two mechanisms—it can modify the wake of the swimmer [15], or the swimmer can benefit from swimming against a flow with lower momentum. In a channel, a flow tank, or a river, the flow near the ground has lower velocity (momentum) compared to the midline. Therefore, we perform the simulations for a quiescent flow to only consider the effect of ground and wake interaction on the swimming performance.

### 2.4. Calculation of Hydrodynamic Forces and Swimming Efficiency

The fish swims along the positive *z* direction. The non-dimensional instantaneous hydrodynamic force coefficient along that direction is calculated as [38]:

(2) $$C_{f}\left(t\right)=\frac{1}{\rho U^{2}L^{2}}\int \left(-pn_{3}+\tau_{3j}n_{j}\right)dA,$$

where dA is the area of an element of the swimmer’s body, $n_{j}$ is the $j^{th}$ component of the unit normal vector on dA, that is, $n_{3}$ is along *z* direction, *p* is the pressure, and $\tau_{ij}$ is the viscous stress tensor. Note that $\tau_{3j}n_{j}$ represents the Einstein’s summation notation. The first term of the integral is the pressure force, and the second term represents the viscous force which are denoted by $C_{p}$ and $C_{v}$, respectively. To calculate the hydrodynamic thrust and drag, respectively, the force contributions are separated into the positive forces (thrust), and the negative forces (drag) as follows [38]:

(3) $$T\left(t\right)=T_{p}+T_{v}=0.5(\int_{A}-pn_{3}dA+|\int_{A}pn_{3}dA|)+0.5(\int_{A}\tau_{3j}n_{j}dA+|\int_{A}\tau_{3j}n_{3}dA\left|\right),$$

(4) $$-D\left(t\right)=-\left(D_{p}+D_{v}\right)=0.5(\int_{A}-pn_{3}dA-|\int_{A}pn_{3}dA|)+0.5(\int_{A}\tau_{3j}n_{j}dA-|\int_{A}\tau_{3j}n_{3}dA\left|\right),$$

where T(t) and D(t) are the dimensional thrust and drag forces respectively, and the subscripts *p* and *v* represent the pressure and viscous terms of the force, respectively. The thrust ($C_{T}$) and drag ($C_{D}$) coefficients are obtained by normalizing T(t) and D(t) with $\rho U^{2}L^{2}$. More details about the calculations of the surface integrals and the accuracy of the methods can be found in Borazjani [39]. The power loss due to the lateral undulations of the fish body is calculated using [38]:

(5) $$P_{side}=\left(\int -pn_{2}\dot{h}dA+\int \tau_{2j}n_{j}\dot{h}dA\right),$$

where $P_{side}$ is the dimensional power loss, and $\dot{h}$ is the velocity of the lateral undulations. The power coefficient ($C_{P_{side}}$) is obtained by normalizing $P_{side}$ with $\rho U^{3}L^{2}$. Using the power coefficient, the Froude propulsive efficiency is [38]:

(6) $$\eta =\bar{C_{T}}\bar{u_{z}}/\left(\bar{C_{T}}\bar{u_{z}}+{\bar{C}}_{P_{side}}\right),$$

where $\bar{C_{T}}$ is the mean thrust coefficient over the swimming cycle, $\bar{u_{z}}$ is the mean swimming speed nondimensionalized by *U*, and ${\bar{C}}_{P_{side}}$ is the mean non-dimensional power coefficient over the swimming cycle.

## 3. Results and Discussion

In this section, the ground effects on the swimming performance (thrust, power and efficiency) of the anguilliform swimmer during steady swimming are discussed in Section 3.1, and the flow field around the anguilliform swimmer during steady swimming, detailing the velocity vectors and vorticity, is discussed in Section 3.2.

### 3.1. Effects of Ground Swimming Performance

**Figure 2:**
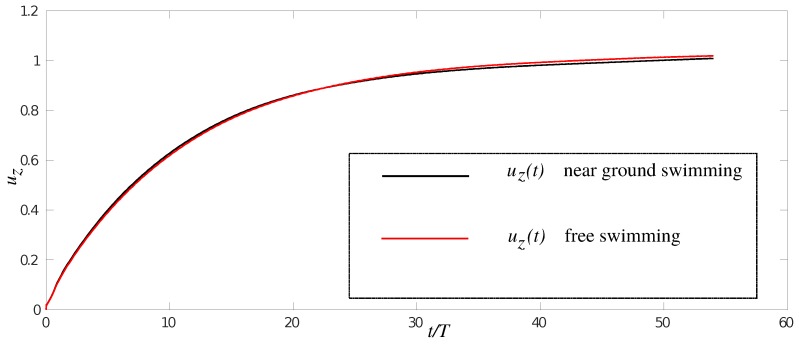
Time history of the non-dimensional velocity of the anguilliform swimmer for near ground and free swimming.

The time history of the anguilliform swimmer’s non-dimensional speed is presented in Figure 2. The swimmer for both cases started from rest, $u_{z}=0$, accelerating until it reached a quasi-steady state at which the mean $u_{z}$ remains constant. This occurs when the thrust produced by the swimmer is equal to the drag it experiences within the fluid during locomotion. The plot of the swimmer’s swimming speed is almost identical for both cases. The steady speed for the free swimming and near ground case is 1.0195 and 1.0165, respectively. In fact, the steady speed of the free swimmer is almost identical for both cases.

**Figure 3:**
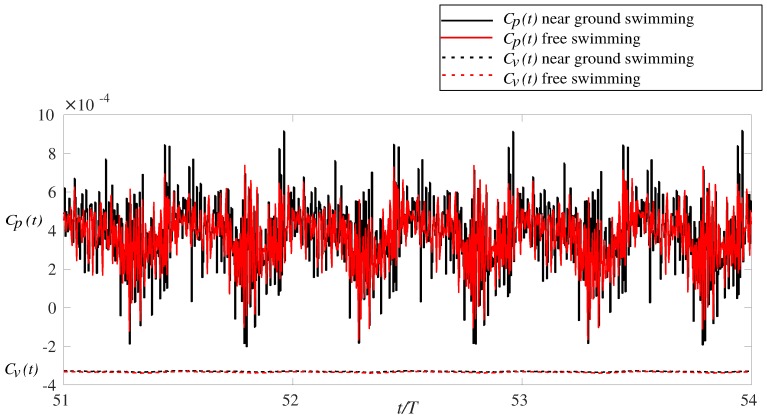
Time history of the pressure force ($C_{p}$) and viscous force coefficient ($C_{v}$) of the anguilliform swimmer swimming for distances: (a) close to the ground (b) at the center; during steady swimming. Positive values indicate thrust force, and negative values indicate drag force.

Figure 3 shows the instantaneous time history of the pressure and viscous force coefficients of the anguilliform swimmer for near ground swimming and free swimming during steady swimming. Based on the thrust and drag definition (Equations (3) and (4)), the positive values of the net force, which is composed of viscous and pressure force (Equation (2)), represent the thrust force and the negative values represent the drag force acting on the swimmer in both cases. As it can be observed in Figure 3, the pressure force is almost positive and the viscous force is always negative during an entire cycle. This indicates that, at almost all instants, the pressure force is the thrust force, and the viscous force is the drag force acting on the swimmer. As shown in Figure 3, the fluctuation of the viscous force is negligible compared to the pressure force.

The mean thrust coefficient does not change by the ground effect, that is, they are $3.78\times 10^{-4}$ and $3.77\times 10^{-4}$, respectively, for free and near ground swimming cases. The noisy behavior of the force in Figure 3 is due to the turbulent nature of the flow. It has been previously seen in anguilliform swimmers in computational simulations for transitional regime [36].

To calculate the swimming efficiency of the anguilliform swimmer, we use the swimming velocity of the swimmer when it becomes quasi-steady. The Froude efficiency is computed by Equation (6). The Froude efficiency for the swimmer at the given Re and St at varying distances from the ground during steady swimming is presented in Table 1. The efficiency for the near ground swimming and free swimming case is 19.92% and 20.73%, respectively, which is practically identical.

The side power required for steady swimming is also reported in Table 1. It can be observed that the mean power for free swimming and swimming near the ground are virtually identical. Therefore, the anguilliform swimmer does not experience any significant increase in the steady speed or performance due to the ground effect. Based on our results, the swimmer had a 0.3% reduction in the speed, 4% reduction in the efficiency, and a 0.3% reduction in the thrust produced near the ground. This shows that there is no significant change in the swimming performance of the swimmer due to the ground effect. Compared to previous work on ground effect, our results match those of Blevins et al. [2] with a robotic model of stingray fins. Our results, however, contradict those of Quinn et al. [10], Fernández Prats et al. [11], Mivehchi et al. [12], Dai et al. [13], Perkins et al. [14], and Kurt et al. [15]. This difference might be due to the direction of the traveling wave’s oscillation. In our case, the traveling wave’s oscillation is parallel to the ground which differs from the cases of Quinn et al. [10], Fernández Prats et al. [11], Mivehchi et al. [12], Dai et al. [13], Perkins et al. [14], and Kurt et al. [15] whose traveling wave’s oscillation was along the ground normal. To investigate why swimming near ground does not improve the anguilliform swimming performance compared to the previous work on rajiform, the flow is examined in the next section.

### 3.2. Flow Field

**Figure 4:**
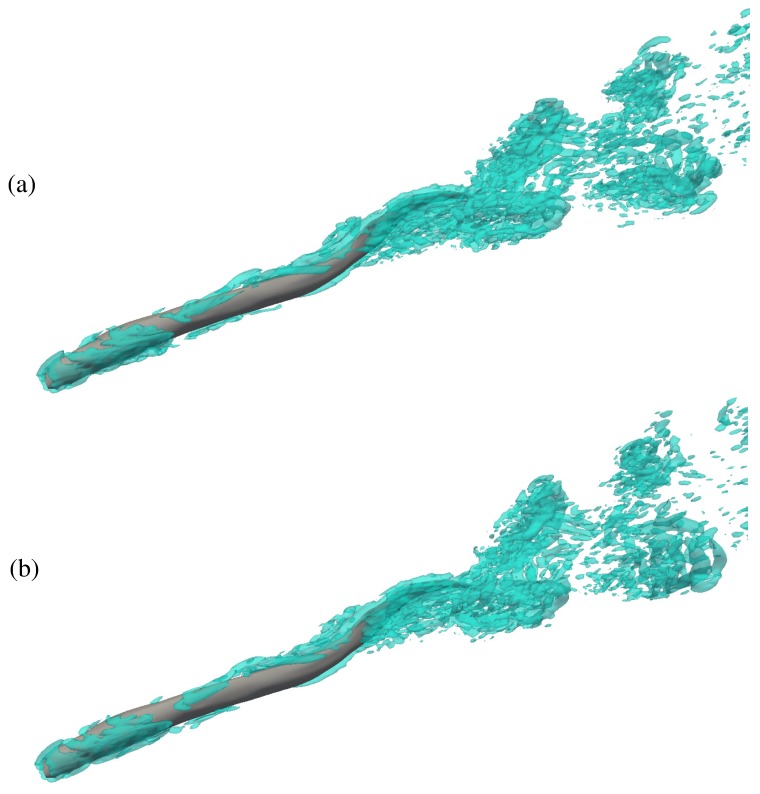
Three-dimensional (3D) vortical structures visualized by the isosurface of q-criterion (Q = 40) showing 3-D wake structures of the anguilliform swimmer swimming (**a**) near ground, and (**b**) freely.

**Figure 5:**
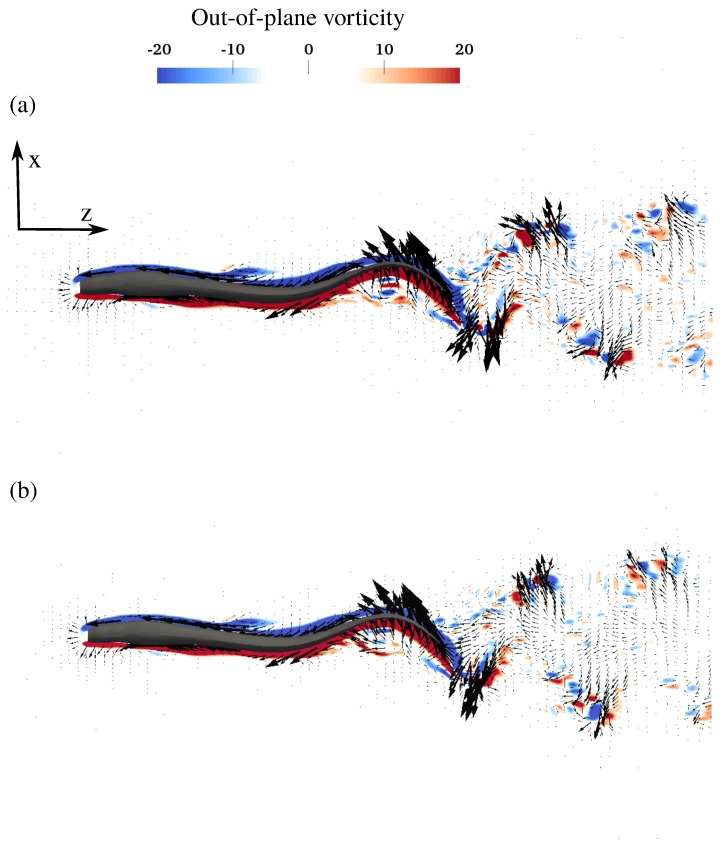
The top view of the instantaneous velocity vectors with vorticity contours of the anguilliform swimmer swimming (**a**) near ground, and (**b**) freely. Only every third vector is plotted for clarity.

**Figure 6:**
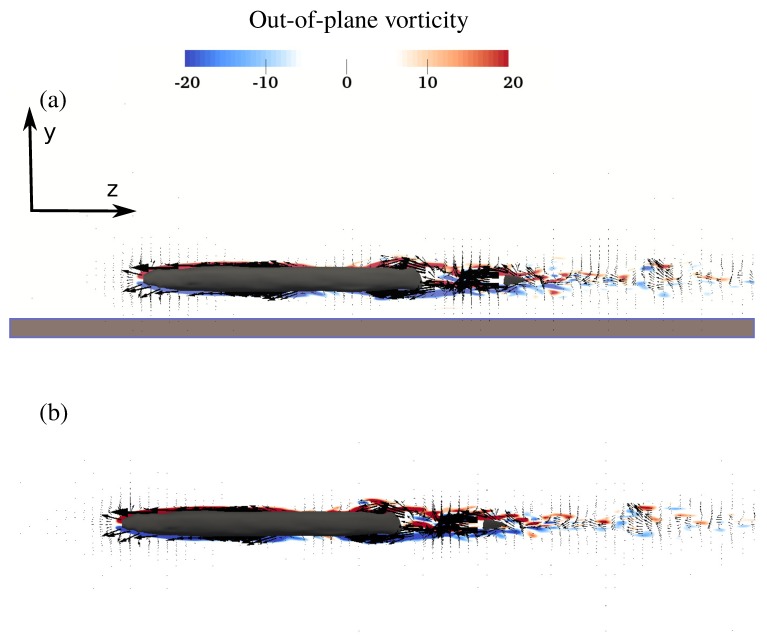
The side view of the instantaneous velocity vectors with vorticity contours of the anguilliform swimmer swimming (**a**) near ground, and (**b**) freely. The brown thick line represents the ground. Only every third vector is plotted for clarity.

The flow field around the swimmers during steady swimming is visualized by the q-criterion, velocity vectors and contours of the out-of-plane vorticity in Figure 4, Figure 5 and Figure 6. Here, Figure 4a,b shows the 3-D structure of the wake of the swimmer by plotting the isosurfaces of the q-criterion [40] for the near ground swimmer and free swimmer, respectively. As it can be observed from Figure 4, the 3-D vortices created are similar for both cases. Figure 5a,b show the flow from the top view for the near ground swimmer and free swimmer, respectively. The swimmers swim with an axial velocity around one in a quiescent flow. Due to no-slip condition, the flow has to go from velocity one (on the fish body) to zero far from the fish. This typically occurs over a thin layer, that is, the boundary layer. The velocity change in such a thin boundary layer generates high velocity gradients. High velocity gradients produce large vorticity in the boundary layer which is observed as a thin high vorticity layer in Figure 5 and Figure 6. As can be observed from Figure 5a,b, both the velocity vectors and vorticity contours near the swimmer are similar for both cases. The velocity vectors adjacent to the swimmer’s body (Figure 5a,b) are high due to the high velocity gradients within the boundary layer. Also, the boundary layer does not separate from the swimmer’s body because of the undulatory motion [32,41] and the wake of the swimmer is turbulent due to high Re. The wake patterns for both cases are similar, that is, the wake structure (Figure 5) is similar to the previous simulations and experiments [1,38,42,43], in which the wake was split laterally and double row patterns were generated. The generation of the double row pattern is observed at high Strouhal numbers [36], which can be viewed as the ratio of the mean lateral tail velocity to the axial swimming velocity. In fact, at high Strouhal numbers, the vortices shed by the swimmer’s tail tend to have a larger lateral velocity component compared to their axial swimming velocity, which transfers the vortices away from the centerline, causing them to spread in the lateral direction.

The effect of near ground swimming can be better observed from the side view visualization (Figure 6). On the free swimmer and near ground swimmer (Figure 6a,b), the velocity vectors are symmetric with respect to the centerline on both sides of the swimmer. The shear layer on both sides of the anguilliform swimmer is similar for both cases. As can be observed from Figure 6a, the shear layer is unaffected by the ground because of the direction of the oscillation and the boundary layer thickness. In fact, the anguilliform swimmer motion does not have any component along the ground normal, according to Equation (1) which makes the wake spread laterally (Figure 5) instead of dorsoventrally (Figure 6). Consequently, the ground does not affect the lateral spreading of the wake in anguilliform swimming at this gap size compared to rajiform swimming. In addition, the boundary layer thickness is small at this Re. In fact, the boundary layer thickness scales with $L/\sqrt{Re}\approx 0.005L$ [44] which is an order of magnitude smaller than the gap size (0.04L). Note that a gap size smaller than the boundary layer’s thickness, for example, gap size <0.005L, may affect the boundary layer, which is not investigated here. Moreover, eels tend to twist their tails when they undulate their bodies, that is, their tails have a motion towards the ground [45,46]. This motion creates a higher vertical velocity (velocity along ground normal) that can increase the wake ground interaction. Nevertheless, investigating the effects of a smaller gap size, in the range of boundary layer thickness, and also the twist of the eel’s tail can be part of a future work.

## 4. Conclusions

The effect of near ground locomotion is studied numerically for an anguilliform swimmer in an initially quiescent flow. The results of simulations indicate that ground effect does not help anguilliform swimmers in quiescent flows for gaps greater than 0.04L. In fact, it is observed that the steady swimming velocity, thrust, power and swimming efficiency of the fish during steady swimming has no significant changes due to the ground effect at this gap size. In addition, the wake of the swimmer is unaffected by the ground, in contrast to the wake of some rajiform swimmers such as stingrays [2], because stingrays undulate themselves along ground normal but eels undulate themselves parallel to the ground. Here, double row vortices that were reported previously [1,36] are created in the wake of the swimmers and the ground does not affect the spreading of the wake. Note that the twisting of the tail of eels which can generate a flow component normal to the ground is not considered in this work.

The boundary layer of the swimmer does not change by the ground effect because the boundary layer thickness of the swimmer is significantly smaller than the gap size. This indicates that neither the wake nor the boundary is affected by the ground in quiescent flow at this gap size. Therefore, anguilliform swimming does not benefit from swimming near ground through the wake interaction mechanism at this gap size, in contrast to rajiform swimming. The ground effect for gaps smaller than 0.04L for which the gap size is similar to the boundary layer thickness or when there is a twist in the tail motion has yet to be investigated. The other mechanism, that is, lower momentum of incoming flow near the ground, still can provide hydrodynamic benefits for swimming near ground to anguilliform swimmers.

## Figures and Tables

**Table 1:** Calculated swimming performances for the anguilliform mode of fish swimming during steady swimming using the computational fluid dynamics (CFD) method. η is the swimming efficiency, ${\bar{C}}_{T}$ is the thrust coefficient, and ${\bar{C}}_{p_{side}}$ is the power coefficient.

Swimming Conditions	η	${\bar{C}}_{T}$	${\bar{C}}_{p_{side}}$
Near ground swimming	19.92%	$3.77\times 10^{-4}$	$1.50\times 10^{-3}$
Free swimming	20.73%	$3.78\times 10^{-4}$	$1.50\times 10^{-3}$

## Abbreviations

The following abbreviations are used in this manuscript:

*a*	amplitude envelope of lateral motion
$a_{max}$	maximum amplitude
*A*	maximum lateral excursion of the tail over a cycle
$C_{f}$	force coefficient
$C_{p}$	pressure force coefficient
${\bar{C}}_{f}$	mean axial force coefficient
$C_{v}$	viscous force coefficient
*D*	drag
*f*	tail-beat frequency
*F*	force exerted on the fish body by the flow
*h*	lateral excursion of the body
$\dot{h}$	velocity of the lateral undulations
*k*	wave number of the body undulations
*L*	fish length
PIV	particle image velocimetry
${\bar{P}}_{side}$	mean power loss
Re	Reynolds number
St	Strouhal number
*t*	time
*T*	thrust
$\bar{T}$	mean thrust force
*U*	swimming speed
*V*	speed of the backward undulatory body wave
*z*	axial flow direction measured along the fish axis from the tip of the fish’s snout
β	slip velocity
η	Froude propulsive efficiency
λ	wavelength
ν	kinematic viscosity of water
ρ	fluid density
τ	tail-beat period
$\tau_{ij}$	viscous stress
ω	angular frequency
